# State ownership, information asymmetry and cash holding: Impact of COVID-19 on Chinese-listed firms

**DOI:** 10.3389/fpsyg.2022.1052979

**Published:** 2022-11-29

**Authors:** Danni Yu, Weini Soh, Bany Ariffi Amin Noordin, Mohamed Hisham Yahya, Badar Latif

**Affiliations:** ^1^Department of International Education, Shandong Youth University of Political Science, Jinan, China; ^2^Department of Accounting and Finance, University of Putra Malaysia, Seri Kembangan, Malaysia

**Keywords:** COVID-19, information asymmetry, cash holdings, state ownership, corporate governance, China

## Abstract

This study investigates the relationship between information asymmetry and cash holdings under the impact of the coronavirus disease 2019 (COVID-19) in China. It likewise explores how state ownership dominates their nexus, particularly during the pandemic. COVID-19 caused increases in cash holdings, and that the information asymmetry’s effect on cash holdings is more pronounced over the COVID-19 period. Additionally, information asymmetry has a weaker effect on corporate cash holdings for state-owned enterprises (SOEs) under the pandemic. Overall, the study shows that state ownership moderates information asymmetry’s impact on cash holdings and softens firms’ precautionary motive for cash holdings during the pandemic.

## Introduction

A large body of theoretical and empirical literature has sought to explain corporate cash holdings in terms of incentives for managers to stockpile cash when markets are imperfect (Opler et al., 1999; Bates et al., 2009; Gao et al., 2013; Amess et al., 2015). Information asymmetry between shareholders and firm managers, a market friction, has always attracted much attention among scholars. Firms prefer financing internally with retained earnings when making decisions on capital structures. These can avoid extra financing costs and reduce the cost resulting from information asymmetry (Myers, 1984; Myers and Majluf, 1984). The “free cash flow theory” is an alternative view demonstrated by Jensen (1986). The theory states that firm managers have managerial discretion on cash augments when severe information asymmetry arises from monitoring difficulties. Consequently, both the pecking order theory and free cash flow theory explain that cash holdings level increases as the information environment exacerbates. Despite the extant empirical literature that has examined the relationship between information asymmetry and corporate cash holding, the nexus during the coronavirus disease 2019 (COVID-19) pandemic has not received much attention from scholars. Furthermore, few empirical studies have explored how state ownership dominates information asymmetry’s effects on corporate cash holdings, especially under exogenous shocks, such as the COVID-19 pandemic. This study extends the analysis by introducing state-ownership heterogeneity and an exogenous shock’s impact on the nexus of information asymmetry and cash holding.

**Figure 1:**
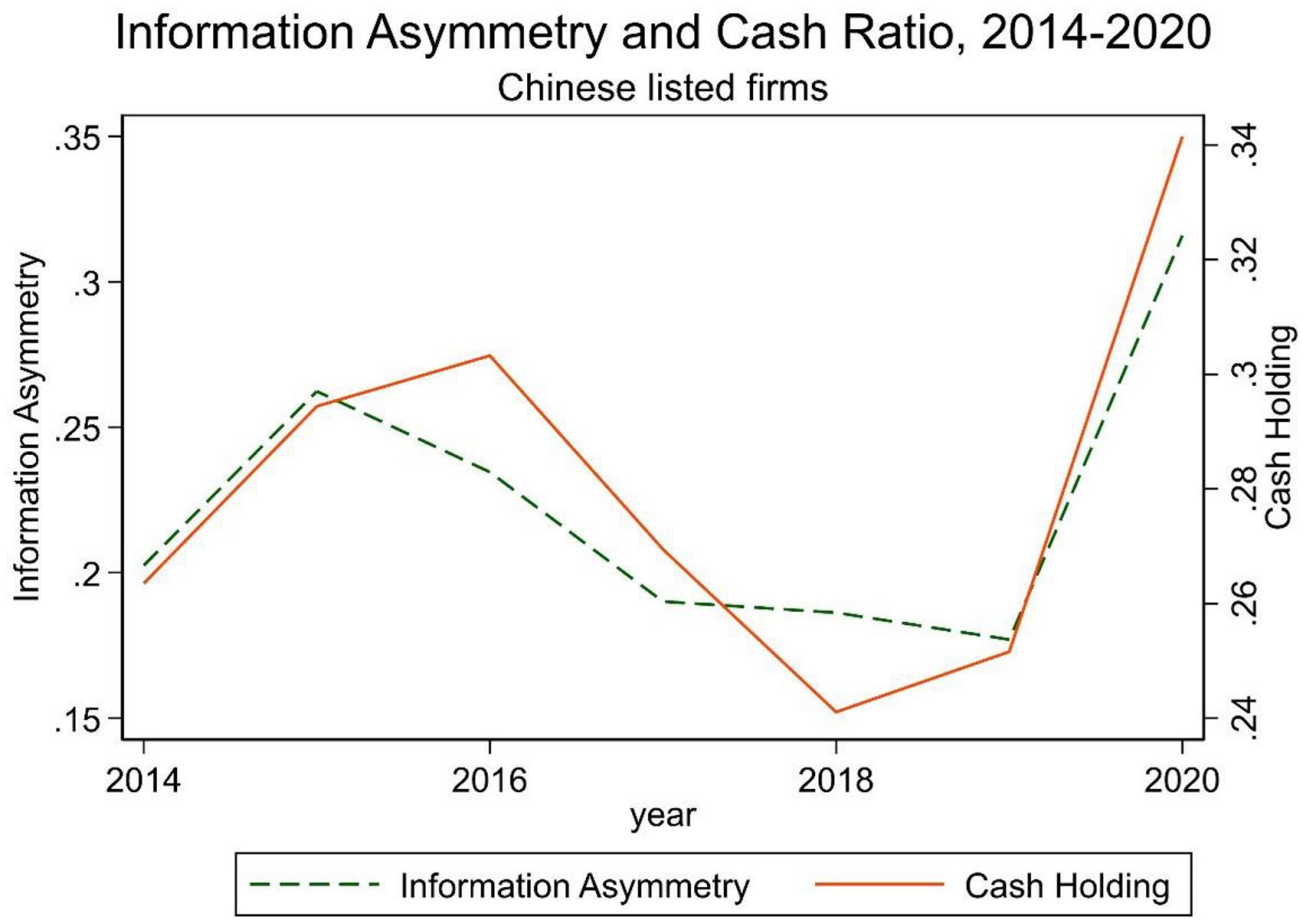
Information asymmetry and cash ratio, 2014–2020.

The COVID-19 outbreak has been an exogenous shock to economies, firms, and individuals. The pandemic brought the world to a standstill and created a devastating crisis that governments could hardly fathom economically or medically, given the previous experience (Li et al., 2021). The crisis put heavy stress on most of the economic activities worldwide and disrupted normal business operations. This has led to firms’ plummeting revenues and cash flows. This study extends the analysis to investigate information asymmetry’s impact on cash holdings by testing the extent to which information asymmetries caused by the COVID-19 outbreak vary. Figure 1 illustrates the linear relationship between information asymmetry and cash holdings. This shows that over the past 7 years, Chinese listed firms’ cash holdings coincide with information asymmetry. They are moving in the same trend all the time, and both hit an all-time peak in the 2020 during the pandemic. Furthermore, cash holdings move after information asymmetry. This indicates that information asymmetry exerts a positive effect on cash holdings. Additionally, information asymmetry increased in 2015, followed by the cash ratio in 2016 due to the 2015–2016 Chinese stock market crash. However, information asymmetry had a secular downward trend after 2015. Thereafter, China revised its securities law, aiming to shift the financial enforcement system from one requiring prior approval and evaluation before the initial public offering (IPO) to a system that requires firms to fully disclose to investors. China did this by enhancing investor protection and increasing penalties for false disclosure or non-disclosure, which should improve market transparency step by step (Blair, 2020). Additionally, we can observe a sharp decline in cash holding from 2016 to 2018. We believe that one of the main reasons comes from the Chinese stock market turbulence which began with the burst of the stock bubble in June 2015 and ended in early 2016. Executives and stockholders will likely hold less cash when the firm has a more predictable future due to improved external markets. Second, the Shenzhen-Hong Kong Stock Connect, a new and direct way for investors to access Chinese capital markets, could contribute to the improvement of market liquidity. A recent study from Huang et al. (2022) examined the effect of stock market liberalization on corporate cash holdings by using China’s Connect programs as quasi-natural experiments. In comparison with non-eligible firms, eligible firms were found to have a sharp decline in cash holdings following the implementation of Connect. Further, Connect programs leads to a reduction in corporate cash holdings through the improvement of the firm’s information environment and the reduction of its financial constraints. Considering this viewpoint, it provides another plausible explanation for the better information environment after 2016.

The state still remains to be an authoritative shareholder in many of China’s largest firms. This leads to a situation in which state-owned enterprises (SOEs) and non-state-owned enterprises (non-SOEs) have become two main identities in China. They are divided based on the firms’ nature as the ultimate controlling shareholder. However, they differ significantly in terms of firms’ ambitions, resource endowments, operational risks, and government regulations (Yang et al., 2017). Since SOEs are often managed by entrenched bureaucrats with political rather than commercial aims, state ownership is connected with the emergence of more complicated agency conflicts, which contributes to poor corporate governance and significant moral hazard problems (Chen et al., 2018). In the absence of good corporate governance, current financial problems could worsen, which alters the incentives to keep cash on hand. Yet, based on the soft budget constraint theory (Kornai, 1979), the government can moderate SOEs’ budget constraints by granting tax discounts, preferential access to credit, and other forms of support. China introduced “COVID-19 prevention and control” bonds for firms to sustain economic growth and to alleviate their financing deficits during the COVID-19 pandemic. However, as of April 21, 2020, SOEs had issued 402 COVID-19 bonds which totals to 317.4 billion yuan, while non-SOEs had only issued 73 bonds which totals to 38.8 billion yuan. This suggests huge gaps in the financing support’s availability between SOEs and non-SOEs during the pandemic.^1^ Consequently, we extend the research by introducing heterogeneity at the firm level (i.e., state ownership). Prior studies have found that regulatory discrimination exists between SOEs and non-SOEs to the extent that SOEs may suffer less from extreme economic shocks. This is due to relatively superior access to financial support, bank credit, and investment opportunities (Yang et al., 2017; Fasano et al., 2022). Therefore, although government ownership results in serious information asymmetry and agency problems, this study hypothesizes that it moderates information asymmetry’s impact on cash holdings. Moreover, it softens the precautionary motive for cash holdings under the impact of the COVID-19 pandemic.

In the present study, we chose China as the case study to explore the nexus among state ownership, information asymmetry, and cash holdings. Firstly, emerging from Wuhan from the early phase to the end of 2020, China experienced almost a complete process from the pandemic’s outbreak to its control. In the aftermath of a black swan event like COVID-19, companies faced immense uncertainty, which may have exacerbated the problem of information asymmetry. Cash holdings of firms are, therefore, more sensitive to information asymmetry. Secondly, the above-mentioned evidence from early 2020 indicates that there is a significant gap between SOEs and non-SOEs in terms of the availability of financing support during the pandemic. In the Chinese context, we believe that the impact of soft budget constraints will be intensified during COVID-19. Finally, as an emerging market, China provides more pronounced information about corporate strategies such as ownership structure. Compared to other countries, the Chinese government has a more significant portion of stakes in listed firms (Choi et al., 2010). According to Jiang and Kim (2020), China’s corporate governance problem is partly related to the country’s concentrated ownership structure. The controlling shareholder holds power through the control of the board of directors and the management of the company as a result of this typical ownership structure. China lacks a number of monitoring mechanisms that are widely accepted as effective in developed countries. This presents an ideal context for studying state ownership’s effects on changes related to information. Collectively, China serves as an ideal context for this study.

This study makes two important contributions to literature. First, we contribute to the extant literature that explores COVID-19’s impact on firm performance (Ashraf, 2020; Shen et al., 2020; Cui et al., 2021; Ding et al., 2021; Li et al., 2021). Second, we contribute to studies that examine information asymmetry’s effect on cash holdings under an exogenous shock’s (e.g., the COVID-19 pandemic) impact and introduce heterogeneity at the firm level (i.e., state ownership). A stream of the previous literature has investigated the impact of information asymmetry between corporate cash holdings (Drobetz et al., 2010; Chung et al., 2015). However, few studies are simply focused on emerging markets like China. While Xiong et al. (2021) explored how corporate cash holdings are impacted by internal information quality in China, their analysis examined information asymmetry within an organization, but we are primarily concerned with the information asymmetry between corporate insiders and outsiders. Most related to our work is the study conducted by Qin et al. (2020). Using the difference-in-differences method, they showed that COVID-19 significantly impacts cash holdings, especially in serious-impact industries. This impact is moderated by goodwill and goodwill impairment during a special period. Based on firm-level data for the first quarter of 2014–2020, their analysis is embodied in the early stages of the COVID-19 outbreak. The present study focuses on the information asymmetry-cash holding nexus and investigates how COVID-19 and state ownership affect their relationship. Additionally, it examines state ownership’s role in influencing the information asymmetry-cash holding nexus under the impact of a pandemic. Moreover, to provide a full picture of COVID-19’s effects, this study examines the same over an extended period that is not limited only to first-quarter data.

The remainder of the paper is organized as follows: Section 2 summarizes the existing literature and provides the research hypotheses. Section 3 presents the research models and the main features of our data. Section 4 reports the results of the empirical analysis. Section 5 presents the conclusion of this study and provides some suggestions for future government policies that may be enforced.

## Theory, literature, and hypothesis

### Information asymmetry, COVID-19, and cash holding

In a perfect capital market, firms can easily obtain access to finance used for operations or investments. However, in actual cases, firms hoard cash for different motives, including transaction, precautionary, and speculative motives (Opler et al., 1999). The COVID-19 pandemic, an unexpected exogenous shock to firms, decreased firms’ returns on investments. Moreover, it increased the difficulty in financing from the outside. During the COVID-19 outbreak, cash served as a buffer for uncertainty when the pandemic deteriorated financial markets’ function. Tawiah and Connor Keefe (2020) found that accumulated cash could help firms alleviate the COVID-19’s adverse impact on investments. Some related studies also provide evidence that firms are inclined to save more when they are under an exogenous shock, such as a financial crisis (Lian et al., 2011; Feng et al., 2022; Jebran et al., 2019; Ashraf, 2020). A recent study from Zheng (2022) proved the precautionary role of cash holdings during the COVID-19 pandemic based on quarterly data from publicly traded US companies. However, his argument relies on the assumption that cash holding is a variable that stands on its own since cash holding serves as an independent variable in his tests. They attribute some firms’ good performance during the Covid-19 period association to the abundant cash reserve. In contrast, cash holding serves as the dependent variable in our study, and we focus on movements in corporate cash holdings themselves. Extant studies lead to our first hypothesis (H1) relating to the nexus of the COVID-19 pandemic and cash holdings. In contrast to Tawiah and Connor Keefe (2020), instead of using data from the first quarter of 2020, we employ panel-fixed estimation using a longer period that extends to the last quarter in 2020. This will provide us a fuller picture of the COVID-19 pandemics effects.

*H1*: COVID-19 is positively related to firms’ level of cash holdings.

According to Fama’s (1970) efficient market hypothesis, investors can obtain the same information as the company’s management without additional costs. However, the information received by everyone is not equal in the real world. It is more realistic to assume that all market participants do not have access to the same information. The COVID-19 pandemic may exacerbate the information asymmetry problem. Business prospects become more uncertain as uncertainty and risk increase during the COVID-19 pandemic. Quarantine policies lead to the cessation of firms’ operational activities and decrease firms’ investments. This is true, particularly in industries such as tourism, catering, and transportation where production, operation, and sales are most affected by the pandemic (Shen et al., 2020). While most firms can barely stay above the water, firms from industries such as those in the medical and accounting fields have increasing revenues despite the pandemic. However, financial statements often reflect speculation and noise during times of high uncertainty. Therefore, firms faced extreme uncertainty when they were first exposed to black swan events like the COVID-19 pandemic. This makes it extremely difficult to predict whether high profits or large losses are sustainable or not. Thus, it results to severe information asymmetry between firm insiders and external investors.

As a result, the COVID-19 pandemic makes it is difficult for investors to obtain precise and timely information on firms. Moreover, firms become more conservative when making investment and financing options. As an important precautionary strategy, firms’ cash holdings change following the information environment’s deterioration. Corporate cash holdings and information asymmetry are tightly interrelated (Drobetz et al., 2010).

The extant literature identifies two main reasons why firms accumulate cash. On the one hand, according to the pecking order theory (Myers, 1984; Myers and Majluf, 1984), firms are inclined to finance internally with retained earnings when making decisions on capital structures. This theory assumes that corporate management is equipped with more information than shareholders. If external financing is needed, they would choose debt first, hybrid securities second, then equity last. The pecking order model’s key element is that asymmetric information costs are related to an increase in external funding. As a result, the decision can avoid extra financing costs and reduce the cost resulting from information asymmetry. Thus, information asymmetry should have a positive effect on corporate cash holdings.

On the other hand, according to Jensen (1986), severe conflicts of interest concerning pay-out policies may arise between shareholders and managers when firms generate plentiful free cash flows. More internal funds, such as firms’ cash holdings, enable firm managers to have more managerial discretion when deciding how to use the funds. Managers are more likely to invest at a lower cost of capital rate or inefficient investments, such as their own pet projects (empire building), instead of paying out this free cash flow to shareholders (Drobetz et al., 2010). Investors equipped with more accurate and valuable information concerning managers’ behaviors are more conducive to monitoring managers’ use of corporate resources (Clarkson et al., 2020). Conversely, the higher information asymmetry resulting from the increased managerial discretion makes managerial actions for shareholders more difficult to monitor and understand. In light of this view, Jensen (1986) suggested that shareholders may restrict managers from accessing free cash flows when there is a higher degree of information asymmetry. Furthermore, Chung et al. (2015) suggested in their monitoring cost hypothesis of cash holdings theory that shareholders may not wish their managers to hold large cash reserves in companies operating under opaque information conditions because monitoring managerial actions are difficult and expensive.

Given the Chinese setting, investor protection regimes and weak corporate governance are insufficient for investors to exert influence over cash holdings. This way, information asymmetry can exacerbate agency conflicts by allowing entrenched managers build cash balances for their own benefits. Although cash is less valuable for a firm in a state of higher information asymmetry, the level of cash holdings increases based on the free cash flow theory (Drobetz et al., 2010; Huang et al., 2014).

This study examines information asymmetry’s impact on the level of corporate cash holdings by conducting research on the extent to which information asymmetries caused by the COVID-19 pandemic vary. On this basis, we believe that information asymmetry’s positive effect on cash holdings is dominant during the COVID-19 pandemic. Moreover, as the pecking order theory suggests, COVID-19 has a stronger influence on information transparency between external investors and internal management. This leads to uncertainties in the business environment and exacerbates firms’ operational risks. Specifically, the COVID-19 pandemic appeared to be an unexpected exogenous shock to the information environment that would raise the level of information asymmetry. Thus, this results to an increase in external financing constraints and leads to an average increase in cash holdings. Consequently, the second hypothesis is:

*H2*: The positive effect of information asymmetry on corporate cash holdings is stronger during the COVID-19 pandemic period.

### State ownership, information asymmetry, and cash holding

According to the soft budget constraint theory (Kornai, 1979), the government can moderate SOEs’ budget constraints by providing tax discounts, preferential access to credit, and other forms of support. Bai et al. (2021) explained that external funds’ formal sources are largely channelled to SOEs. Even as discussed by Lazzarini and Musacchio (2018), firms that have reformed corporate governance by state capital are less responsive to adverse shocks that require rapid adjustment than private firms. Nevertheless, when facing difficulties in raising funds, non-SOEs can only turn to alternative ways. Prior studies have shown that the motives for holding cash should be less relevant for SOEs because of the lower cost of raising external funds. Megginson et al. (2014) found that the level of cash holdings rises as state ownership declines using a sample of 2065 Chinese firms over the period 2000–2012. Yang et al. (2017) showed that cash holdings contribute to firms’ investment efficiencies with regard to tight monetary policies. This type of monetary policies is especially prominent for non-SOEs, not SOEs. Theoretically, asymmetric information costs related to the increase in external funding, which is described in the pecking order theory, are less important for SOEs than for non-SOEs.

Moreover, based on the agency theory (Chen et al., 2018), SOEs tend to be more inefficient than non-SOEs as a result of the separation of ownership and control. Hsu and Liao (2022) shows that through better monitoring and therefore less information asymmetry, a good corporate governance framework may mitigate the impact of COVID-19 on stock markets. Although government ownership can lead to serious information asymmetry problems, governments lack a strong and active monitoring mechanism. Furthermore, they are mostly evaluated with political goals instead of profit maximization. In this sense, the information asymmetry resulting from an increase in managerial discretion would have a weaker impact on shareholders for SOEs than on shareholders for non-SOEs. Moreover, existing studies have found that state ownership appears to give rise to conservative financial policies aimed at promoting stability (Jaslowitzer et al., 2018). In times of high uncertainty such as during the COVID-19 pandemic, conservative and stability-seeking policies and preferential treatments SOEs benefited from helped them deal with extreme situations smoothly. This reasoning brings about the third hypothesis:

*H3*: The effect of information asymmetry on corporate cash holdings is weaker among state-owned firms, even during the COVID-19 pandemic.

## Data collection and variable measurement

Our sample consisted of firm-level data from 2014 to 2020 that were obtained from the China Stock Market & Accounting Research (CSMAR). We selected non-financial and non-insurance-listed companies on the Shanghai and Shenzhen stock exchanges. China was selected as the study sample because it experienced almost a complete process from the pandemic’s outbreak up to its control in 2020. The following samples were excluded to harmonize the research objects: (1) firms that have undergone special treatment (ST), *ST, or have been delisted; (2) firms in the financial insurance industry; and (3) firms with significant missing data. After screening, 2,751 companies were included in the study. We winsorized the data at the 0.01 and 0.99 levels to remove outliers and minimize data bias.

### Measurement of information asymmetry

Our main proxy is the analysts’ forecast error (ASYER). It is the difference between analysts’ forecasts per share and the actual earnings per share for the fiscal year. The forecast errors of analysts can be a reflection of the information environment in which they operate. According to Barron et al. (1998), analysts’ forecast errors can be divided into common and idiosyncratic error components, which arise from errors in public information and in the private information analysts rely upon, respectively. In comparison with other measures of information asymmetry, analysts’ forecasts are more directly related to the information environment and have significant informative content. Moreover, the analysts’ forecasts also include information on possible risk factors under exogenous shocks, such as COVID-19.

According to Elton et al. (1982), analysts’ errors decline monotonically as the end of the fiscal year approaches. Moreover, most of the forecast errors made in the last month of the fiscal year are not industry errors nor are they caused by other macroeconomic factors. Rather, they can be explained by the misestimation of firm-specific characteristics. A higher level of error in analysts’ forecasts indicates a higher level of information asymmetry. The formula is as follows:


$$\text{ASYER}=\text{ln}(1+\frac{\left|EPSforcast-EPSactual\right|}{\left|median\;EPS\right|}).$$

Here, the EPS forecast refers to the average of all analysts’ forecasts for a firm. Based on the studies conducted by Drobetz et al. (2010) and Elton et al. (1982), the present study computes the forecast value in December to minimize industry-and country-specific factors’ impact. Additionally, this formula is also scaled by the median value to make it comparable among various firms in different industries. We also use the dispersion of analysts’ forecasts (ASYDISP) and a dummy variable (ASYDUMMY) which takes a value of 1 if a firm exhibits a value of ASYDISP above its industry median in a given year. Otherwise, it takes a value 0 as the measure of information asymmetry based on prior studies (Drobetz et al., 2010; Fosu et al., 2016). The results are robust to information asymmetry’s alternative measures. For brevity, we did not report the regression results anymore.^2^

### Empirical methodology

To examine hypotheses 1 and 2 (H1 and H2), we developed the following research models to examine the COVID-19’s impact on corporate cash holdings and on the relationship between information asymmetry and corporate cash holdings. For Model 1, we included the COVID dummy variable as the main independent variable. For Model 2, we added an interactive term between information asymmetry and the COVID dummy to the research model to investigate the relationship between information asymmetry and corporate cash holdings during the COVID-19 pandemic. The models are as follows:


$$\begin{array}{c} Cash_{i,t}=\gamma_{0}+\gamma_{1}COVID+\gamma_{2}SIZE_{i,t}+\gamma_{3}LEV_{i,t}\\ +\gamma_{4}TANGIBILITY_{i,t}+\gamma_{5}TURNOVER_{i,t}\\ +\gamma_{6}MTB_{i,t}+\gamma_{7}CF_{i,t}+\gamma_{8}NWC_{i,t}\\ +\gamma_{9}CAPX_{i,t}+\gamma_{10}SOE_{i,t}+\gamma_{11}IND+\varepsilon_{i,t} \end{array}$$

                  (Model 1)


$$\begin{array}{c} Cash_{i,t}=\alpha_{0}+\alpha_{1}ASYER_{i,t}+\alpha_{2}COVID+\alpha_{3}ASY*COVID\\ +\alpha_{4}SIZE_{i,t}+\alpha_{5}LEV_{i,t}+\alpha_{6}TANGIBILITY_{i,t}\\ +\alpha_{7}TURNOVER_{i,t}+\alpha_{8}MTB_{i,t}+\alpha_{9}CF_{i,t}\\ +\alpha_{10}NWC_{i,t}+\alpha_{11}CAPX_{i,t}+\alpha_{12}SOE_{i,t}+\alpha_{13}IND+\varepsilon_{i,t} \end{array}$$

                  (Model 2)

Our third hypothesis (H3) attempts to test state ownership’s impact on corporate cash holdings. Moreover, it tests its impact on the relationship between information asymmetry and corporate cash holdings. We tested H2 by including an interaction term between information asymmetry and SOE. The model is as follows:


$$\begin{array}{c} Cash_{i,t}=\beta_{0}+\beta_{1}ASYER_{i,t}+\beta_{2}ASYER*SOE\\ +\beta_{3}SIZE_{i,t}+\beta_{4}LEV_{i,t}+\beta_{5}TANGIBILITY_{i,t}\\ +\beta_{6}TURNOVER_{i,t}+\beta_{7}MTB_{i,t}+\beta_{8}CF_{i,t}\\ +\beta_{9}NWC_{i,t}+\beta_{10}CAPX_{i,t}+\beta_{11}SOE_{i,t}\\ +\beta_{12}IND+\beta_{13}YEAR+\varepsilon_{i,t} \end{array}$$

                  (Model 3)

Here, iandt denote firm and year, respectively. The dependent variable $Cash_{i,t}$ pertains to the cash ratio, which represents the firm’s cash holdings level. Although the cash-to-asset ratio is the most widely used traditional measure in prior studies, we found that it generates bias due to the COVID-19 pandemic’s impact on assets. Thus, we use the cash ratio to capture the change in the corporate cash holdings’ level based on the study by Qin et al. (2020). Following prior research (Opler et al., 1999; Gao et al., 2013; Lei et al., 2018), the regressions include several control variables to reduce the interference from confounding factors. These variables include firm size (SIZE), leverage (LEV), tangible asset ratio (TANGIBILITY), asset turnover ratio (TURNOVER), market-to-book ratio (MTB), cash flow ratio (*CF*), net working capital ratio, and capital expenditures (CAPX). The firm size (SIZE) has been controlled since larger firms may have less information asymmetry, resulting in easier access to external financing and a reduction in the requirement for large cash reserves (Drobetz et al., 2010). Leverage (LEV) is included in this study since the factors that make debt costly are often factors that make cash holdings advantageous. Our controls also include current liquidity levels, such as cash flows (*CF*), working capital (NWC), and turnover ratios (TURNOVER), since firms may elect to hold liquid assets in addition to cash in order to protect themselves from losses. The market-to-book ratio (MTB) is often used as a proxy for investment opportunities. Based on Opler et al. (1999), firms with high market-to-book ratios could be expected to hold more cash since they will incur higher costs if their financial condition worsens. Additionally, companies with low asset tangibility (TANGIBILITY) are forced to accumulate precautionary savings due to the high cost of external financing (Lei et al., 2018). Capital expenditure (CAPX) is also controlled according to the static trade theory, in which firms with more capital expenditures have more liquid assets. (Opler et al., 1999). The definitions of these variables are presented in Table 1.

**Table 1:** Description of variables.

Variables	Names	Definitions
CASH	Cash ratio	The main proxy for level of corporate cash holding
ASYER	Analysts’ forecast error	The main proxy for information asymmetry; it is calculated by obtaining the difference between analysts’ forecast per share and the actual earnings per share for the fiscal year.
COVID	Covid dummy	This is equal to 1 if during the COVID-19 period in 2020; otherwise, it is equal to 0.
SIZE	Firm size	The natural logarithm of the book value of total assets net of cash.
LEV	Leverage	The ratio of long-term debt plus debt in current liabilities to total assets.
TANGIBILITY	Tangibility	The ratio of tangible assets to total assets.
TURNOVER	Turnover	The ratio of operating expenses to total assets.
MTB	Market to book ratio	The ratio of total assets less the book value of common equity, plus the total market value of equity, all divided by the total book assets.
*CF*	Cash flow	The ratio of operational cash flows to the total assets.
NWC	Net working capital	The ratio of net working capital to the total assets.
CAPX	Capital expenditure	The ratio of capital expenditures to the book value of the total assets.
SOE	State-owned-enterprise	An indicator variable that takes a value of 1 if it is a state-owned enterprise; otherwise, it is 0.
IND	Industry fixed effect	This is used to represent industry-fixed effects.
YEAR	Year fixed effect	This is used to represent year-fixed effects.

In order to determine whether our estimates are sensitive to the bias caused by omitted variables, we conducted a sensitivity test using the package sensemakr in Stata (Cinelli and Hazlett, 2020). The Robustness Value (RV) is calculated to determine how much residual variance for the treatment (COVID) and outcome (CASH) the omitted unobserved factor should explain to achieve a zero estimate of the treatment coefficient (COVID). This result has an RV of 0.09, indicating that the unobservable that produced this result explains 9% of the residual COVID and CASH variance. For an omitted variable, this is a relatively high percentage. Since our specification follows previously published work and is theoretically based, we maintain that such important confounders were unlikely to be omitted during the development of our baseline model. We apply panel data regression to test the hypotheses given that our sample includes cross-sectional and time-series data (Wooldridge, 2018). We estimate our hypothesis using panel-fixed estimation based on the Hausman test (Hausman, 1978), with a chi-square of 207.59 and a value of p less than 0.0001. We also employ a pooled OLS regression for the robustness checks.

## Empirical results

### Descriptive statistics, correlations and discussion of The results

Table 2 reports the descriptive statistics of the variables for the model. Panel A shows that the dependent variable ranges from 0.033 to 2.286. The mean and median values were 0.402 and 0.277, respectively. The mean and median values of ASYER were 0.328 and 0.228, respectively. The mean value of 0.335 for SOE shows that 33.5% of the sample firms are state-owned firms.

**Table 2:** Summary of the statistics.

Variable	Mean	S.D.	Min	Median	Max
Panel A: Firm-specific data					
CASH	0.402	0.392	0.0330	0.277	2.286
ASYER	0.328	0.354	0	0.228	2.080
SOE	0.335	0.472	0	0	1
SIZE	22.63	1.324	20.33	22.41	26.61
LEV	0.424	0.193	0.0670	0.416	0.851
TANGIBILITY	0.908	0.106	0.488	0.947	1
TURNOVER	16.30	67.05	0.168	3.555	574.6
MTB	2.147	1.370	0.851	1.713	8.632
*CF*	0.0530	0.0660	−0.123	0.0510	0.239
NWC	0.223	0.225	−0.323	0.221	0.730
CAPX	0.228	0.170	0.00400	0.192	0.735
Panel B: Descriptive statistics of different periods												
Variable		2014–2020			2015–2017			2018–2019			2020	
		Mean		S.D.	Mean		S.D.	Mean		S.D.	Mean		S.D.
CASH	0.402	0.392	0.416	0.404	0.335	0.302	0.501	0.471
ASYER	0.328	0.354	0.340	0.378	0.291	0.341	0.381	0.296
SOE	0.335	0.472	0.345	0.475	0.316	0.465	0.296	0.456
SIZE	22.63	1.324	22.52	1.283	22.80	1.335	22.89	1.341
LEV	0.424	0.193	0.420	0.198	0.430	0.184	0.426	0.183
TANGIBILITY	0.908	0.106	0.903	0.112	0.907	0.105	0.909	0.100
TURNOVER	16.30	67.05	15.27	62.49	19.34	76.01	14.80	66.93
MTB	2.147	1.370	2.370	1.423	1.720	1.046	2.335	1.724
*CF*	0.0530	0.0660	0.0500	0.0660	0.0670	0.0640	0.0370	0.0630
NWC	0.223	0.225	0.226	0.228	0.212	0.219	0.229	0.213
CAPX	0.228	0.170	0.231	0.174	0.229	0.168	0.195	0.149
Panel C. Selected firm-level data of SOEs and non-SOEs												
	Non-state-owned firms (66.5% of the whole sample)						State-owned firms (33.5% of the whole sample)					
Variables	Before year 2020			Year 2020			Before year 2020			Year 2020			
	N	Mean	S.D.	N	Mean	S.D.	N	Mean	S.D.	N	Mean	S.D.
CASH	6,547	0.403	0.393	1,125	0.487	0.453	3,386	0.353	0.336	472	0.534	0.512
ASYER	6,547	0.318	0.344	1,125	0.391	0.295	3,386	0.322	0.394	472	0.358	0.298

This table shows descriptive statistics for the variables. Panel A presents descriptive statistics for the whole sample from 2014 to 2020: mean, standard deviation (S.D.), min, and median. Panel B describes descriptive statistics in different periods. Panel C shows descriptive statistics (N, mean, S.D.) for selected firm-level variables of SOEs and non-SOEs. All presented variables are winsorized at 1 and 99% levels. Variables are defined in Table 1. This table presents correlations matrices for the variables used in the main regressions. ***, **, and * denote statistical significance at the 1%, 5%, and 10% levels, respectively. Variables are defined in Table 1.

Furthermore, Panel B reports the variables’ descriptive characteristics over different periods from 2014 to 2020. We divided the sample into several periods to present the variables’ descriptive characteristics. This was done avoid interference from the 2015–2016 Chinese stock market crash and shed light on the COVID-19 pandemic’s impact. According to Panel B, the cash ratio and information asymmetry increased from 2015 due to the Chinese stock market crash’s impact. However, it experienced a downward trend from 2018 to 2019. Nonetheless, they both hit a peak during the COVID-19 pandemic. In sum, firms hoard much more cash and have a higher information asymmetry, larger firm size, and higher net working capital ratio during the COVID-19 pandemic. Moreover, firms also have lower turnover, cash flow, and capital expenditure ratios.

Panel C presents a summary of the dependent variable CASH and information asymmetry measures for SOEs and non-SOEs before and during the COVID-19 pandemic. The mean value of 0.353 for CASH, compared with the values of 0.220 and 0.322 for the two information asymmetry measures of SOEs, shows that the cash SOEs hoard is less than that of non-SOEs before 2020. This is true even though SOEs have higher information asymmetry.

The study also considers the issue of multicollinearity, and the calculated correlation results are presented in Table 3. The table reveals that the information asymmetry measure and COVID are all negatively correlated with CASH. Furthermore, state ownership (SOE) was significantly negatively correlated with CASH. The results further indicate that there is a modest correlation among the variables and that multicollinearity does not turn out to be an issue in the analysis of the study. The symbols *, **, and *** indicate significance at the 10, 5, and 1% levels, respectively.

**Table 3:** Correlation matrix.

	CASH	COVID	ASYER	SIZE	LEV	TANGIB~Y	TURNOVER	MTB	*CF*	NWC	CAPX	SOE
CASH	1											
COVID	0.101***	1										
ASYER	0.061***	0.060***	1									
SIZE	−0.116***	0.079***	−0.054***	1								
LEV	−0.246***	0.004	0.121***	0.581***	1							
TANGIBILITY	0.001	0.006	−0.032***	0.086***	0.148***	1						
TURNOVER	0.041***	−0.009	0.000	0.010	−0.034***	−0.066***	1					
MTB	0.149***	0.055***	−0.002	−0.436***	−0.378***	−0.004	0.010	1				
*CF*	−0.068***	0.095***	−0.205***	−0.010	−0.204***	0.019**	0.065***	0.136***	1			
NWC	0.368***	0.012	−0.138***	−0.426***	−0.629***	0.164***	−0.052***	0.328***	−0.023**	1		
CAPX	−0.268***	0.079***	0.036***	0.112***	0.052***	0.189***	0.051***	−0.130***	0.286***	−0.519***	1	
SOE	−0.049***	0.033***	−0.004	0.439***	0.283***	0.163***	0.024**	−0.201***	−0.004	−0.258***	0.229***	1

This table presents correlations matrices for the variables used in the main regressions. ***, **, and * denote statistical significance at the 1%, 5%, and 10 % levels, respectively. Variables are defined in Table 1.

### Regression results

#### The impact of COVID-19 On corporate cash holdings

As shown in Table 4, this study tests COVID-19’s impact on corporate cash holdings by including the COVID dummy variable. The adjusted R-square ranges from 0.1687 to 0.2430. This indicates that the variables included in the model explain approximately 16.87 and 24.30% of the variations in CASH. The COVID dummy variable’s coefficients are positive and statistically significant at the 1% level for both the FE model and OLS model. This finding suggests that COVID-19 has a positive effect on firms’ level of cash holdings. The coefficients of the COVID dummy variable are 0.0930 for the OLS model and 0.105 for the FE model. This suggests that the COVID-19 pandemic is associated with a remarkable 23.13 to 26.12% increase in the corporate cash holdings ratio.^3^ These are consistent with Hypothesis 1.

**Table 4:** The impact of COVID-19 on corporate cash holdings.

Variables	OLS MODEL 1	FE MODEL 1
Constant	0.0467	0.177**
	(0.59)	(2.17)
COVID	0.0932***	0.105***
	(7.91)	(9.28)
SOE	0.0538***	0.0234***
	(6.74)	(3.07)
SIZE	0.0185***	0.00855**
	(5.46)	(2.52)
LEV	−0.215***	−0.249***
	(−5.62)	(−6.77)
TANGIBILITY	−0.0749*	0.0477
	(−1.69)	(1.10)
TURNOVER	0.000336***	0.00000760
	(4.49)	(0.10)
MTB	0.0113***	0.00926***
	(3.43)	(2.83)
*CF*	−0.314***	−0.249***
	(−5.43)	(−4.51)
NWC	0.483***	0.462***
	(13.21)	(13.06)
CAPX	−0.252***	−0.175***
	(−7.55)	(−5.20)
_cons	0.0467	0.177**
	(0.59)	(2.17)
Industry_FE	NO	Yes
N	11,415	11,415
adj. R2	0.168	0.243

This table reports the results of the impact of COVID-19 corporate cash holdings. The values in parentheses represent t-statistics. Column (2) controls for industry fixed effect. ***, **, and * denote statistical significance at the 1, 5, and 10% levels, respectively. Variables are defined in Table 1.

#### Information asymmetry and corporate cash holdings — Impact of COVID-19

In Table 5, this study tests COVID-19’s impact on the relationship between information asymmetry and corporate cash holdings by including the COVID dummy variable and its interaction term with information asymmetry. The adjusted R-square ranges from 0.1821 to 0.2545. This indicates that the variables included in the model explain approximately 18.21 and 25.45% of the variations in CASH. The coefficients of ASYER have positive signs and are significant at the 1% level for both models. This indicates a positive relationship between information asymmetry and corporate cash holdings. Furthermore, we likewise find support for the second hypothesis. The interaction term between ASYER and COVID is highly, statistically, and economically significant. The marginal effect of ASYER during the COVID-19 year is 0.284 for the OLS model and 0.241 for the FE model. On the other hand, before the COVID-19 year, the marginal effect of ASYER Is 0.106 for the OLS model and 0.102 for the FE model.^4^ This indicates that, *ceteris paribus*, one standard deviation increase in ASYER generates a 21.22 to 25% gain in CASH in the COVID-19 year. However, there is only a 9 to 9.33% increase in pre COVID-19 years.^5^ Thus, the information asymmetry measured by ASYER in the COVID-19 year exerts up to 16% more impact on corporate cash holdings than in pre-COVID-19 years. This is consistent with the view that information asymmetry’s positive effect on corporate cash holdings is stronger during the COVID-19 pandemic period. In other words, the COVID-19 pandemic may have compounded the problem of information asymmetry. This causes firm cash holdings to be more sensitive to information asymmetry.

**Table 5:** Information asymmetry and corporate cash holdings — impact of COVID-19.

Variables	OLS MODEL 2	FE MODEL 2
Constant	−0.1250	−0.0488
	(−1.54)	(−0.63)
ASYER	0.1062***	0.1026***
	(9.86)	(9.76)
ASYERXCOVID	0.1783***	0.1382***
	(4.06)	(3.49)
COVID	0.0187*	0.0456**
	(1.02)	(2.59)
SOE	0.0533***	
	(6.71)	
SIZE	0.0242***	0.0139***
	(7.09)	(5.22)
LEV	−0.2360***	−0.2741***
	(−6.19)	(−7.45)
TANGIBILITY	−0.0663	0.0498
	(−1.50)	(1.43)
TURNOVER	0.0003***	−0.0000
	(4.44)	(−0.10)
MTB	0.0108***	0.0090***
	(3.30)	(2.86)
*CF*	−0.1700***	−0.1161**
	(−2.98)	(−2.26)
NWC	0.5090***	0.4850***
	(13.97)	(13.85)
CAPX	−0.2614***	−0.188***
	(−7.86)	(−5.38)
Industry_FE	NO	Yes
N	11,415	11,415
adj. R2	0.1687	0.2430

This table reports the results of the impact of COVID-19 on information asymmetry-cash holding nexus. The values in parentheses represent t-statistics. Column (2) controls for industry fixed effect. ***, **, and * denote statistical significance at the 1, 5, and 10% levels, respectively. Variables are defined in Table 1.

#### Information asymmetry and corporate cash holdings — The role of state ownership

As mentioned earlier, information asymmetry’s effect on corporate cash holdings may vary across ownership structures. The descriptive statistics of variables for SOEs and non-SOEs in Table 2 show that although SOEs have higher information asymmetry, the cash they hoarded is less than non-SOEs before 2020. Thus, we expect that the effect of information asymmetry on corporate cash holdings is weaker among SOEs in our setting.

This study tests this hypothesis by including an interaction term between information asymmetry and firm ownership dummies. The results in Table 6 show that the coefficient between the measure of information asymmetry and SOE dummies and their interaction terms are all significant for the OLS and FE models. Furthermore, the coefficients of ASYER are all positive and significant at the 1% level for the OLS and FE models. Specifically, the marginal effects of ASYER are 0.0878 for the OLS model and 0.0923 for the FE model of SOEs. On the other hand, it is 0.1573 for the OLS model and 0.1260 for the FE model of non-SOEs. Economically, the estimate indicates that, *ceteris paribus*, a one standard deviation increase in ASYER is associated with a 7.73 to 8.12% increase in CASH for SOEs. Conversely, it is associated with an increase of 11 to 13.8% in CASH for non-SOEs. Taken together, these results are consistent with Hypothesis 2. It confirms that information asymmetry’s effect on corporate cash holdings is weaker among SOEs. This can be theoretically explained in two ways. First, cash is less relevant for SOEs as a precautionary motive because banks are willing to extend credit. This is true particularly when firms are in states with severe information asymmetry, such as when they are financially distressed or lack access to external private financing. Second, the government can moderate SOEs’ budget constraints, which exaggerates agency problems, where managers may reserve more cash to invest in political-driven projects rather than NPV-maximizing projects. Therefore, it is necessary for the government to develop or enhance rules and regulations related to disclosures and transparency within SOEs. A good governance of SOEs will provide information that is of value to outsiders.

**Table 6:** Information asymmetry and corporate cash holdings — role of state ownership.

Variables	OLS MODEL 3	FE MODEL 3
Constant	−0.2258***	−0.0030
	(−2.78)	(−0.04)
ASYER	0.1573***	0.1260***
	(10.70)	(8.99)
ASYERXSOE	−0.0692***	−0.0337*
	(−3.31)	(−1.69)
SOE	0.0708***	0.0284***
	(7.21)	(3.06)
SIZE	0.0286***	0.0158***
	(8.35)	(4.61)
LEV	−0.2620***	−0.2879***
	(−6.84)	(−7.88)
TANGIBILITY	−0.0488	0.0673
	(−1.11)	(1.57)
TURNOVER	0.0003***	0.0000
	(4.43)	(0.20)
MTB	0.0125***	0.00249
	(3.88)	(0.72)
*CF*	−0.2198***	−0.0651
	(−3.84)	(−1.17)
NWC	0.4947***	0.4778***
	(13.58)	(13.66)
CAPX	−0.2796***	−0.2120***
	(−8.41)	(−6.34)
Industry_FE	NO	Yes
Year_FE	NO	Yes
N	11,415	11,415
adj. R2	0.1749	0.2578

This table reports the results of the impact of state ownership on information asymmetry-cash holding nexus. The values in parentheses represent t-statistics. Column (2) controls for industry×year fixed effect. ***, **, and * denote statistical significance at the 1, 5, and 10% levels, respectively. Variables are defined in Table 1.

#### Information asymmetry, corporate cash holding and COVID-19: SOEs vs. non-SOEs

We extend the analysis by splitting the sample into different subgroups: SOEs and non-SOEs. We likewise use firm-fixed effect estimation to investigate the COVID-19 pandemic’s impact on cash holdings and look into the relationship between such an impact with information asymmetry for each group. Table 7 shows that the coefficient of the interaction term between ASYER and the COVID dummy variable is positive and significant at the 1% level for non-SOEs. However, it is positive but insignificant for SOEs. Additionally, most of the coefficients of the COVID dummy variable and ASYER are statistically significant for both SOEs and non-SOEs. The adjusted R-square ranges from 0.6490 to 0.7264. This indicates that the variables included in the model explain approximately 64.90 and 72.64% of the variations in CASH for the two subsamples. Finally, a Chow test was conducted to investigate whether the coefficients are significantly different for SOEs and non-SOEs.

**Table 7:** Information asymmetry, corporate cash holdings and COVID-19: SOEs vs. non-SOEs.

Variables	SOE	NON-SOE
Constant	0.0816	−0.5893*
	(0.18)	(−1.93)
ASYER	0.0313**	0.0420***
	(2.49)	(3.17)
ASYERXCOVID	0.1062	0.1752***
	(1.57)	(3.79)
COVID	0.1265***	0.0313*
	(4.85)	(1.69)
SIZE	0.0089	0.0320***
	(0.57)	(2.59)
LEV	0.1399*	0.1689**
	(1.70)	(2.47)
TANGIBILITY	−0.0416	0.0338
	(−0.22)	(0.38)
TURNOVER	0.0000	−0.0004**
	(0.44)	(−2.25)
MTB	−0.0030	−0.0061
	(−0.38)	(−1.61)
*CF*	0.1660*	0.0205
	(1.86)	(0.30)
NWC	0.6185***	1.0261***
	(8.01)	(16.95)
CAPX	−0.2496***	−0.4272***
	(−3.07)	(−5.50)
Industry_FE	Yes	Yes
Chow test	12.82***	
N	3,846	7,569
adj. R2	0.7264	0.6490

This table reports the results of the impact of COVID-19 on information asymmetry-cash holding nexus under different samples. We split the sample into different subgroups: SOEs and non-SOEs. The values in parentheses represent t-statistics. All regressions control for industry fixed effect. A Chow test was conducted to investigate whether the coefficients are significantly different for SOEs and non-SOEs. ***, **, and * denote statistical significance at the 1, 5, and 10% levels, respectively. Variables are defined in Table 1.

The results demonstrate that information asymmetry has a weaker effect on corporate cash holdings on SOEs during the COVID-19 pandemic than on non-SOEs. More importantly, the results show that the COVID-19 pandemic has a greater impact on cash holdings for SOEs than for non-SOEs. This is consistent with the previous studies done Chen et al. (2018) and Jaslowitzer et al. (2018), which investigated the role of financial crises in the relationship between state ownership and cash policy. These studies found that SOEs tend to formulate financial policies such as holding more cash to improve stability, especially during periods of crisis.

### Robustness checks

Additionally, the study offers an additional analysis to mitigate endogeneity issues and corroborate the results. According to Chung et al. (2015), it is likely that information asymmetry can be determined by corporate cash holdings. We address this concern by using changes in the variables as a robustness check. Specifically, the study employs yearly changes in dependent variables to verify whether they can be explained by contemporaneous and lagged changes in information asymmetry. The results are presented in Table 8. Consistent with our hypothesis, the estimations yield supportive results after accounting for potential endogeneity issues, as in the previous regressions.

**Table 8:** Robustness checks using changes in the variables.

Variables	MODEL 1	MODEL 2	MODEL 3
Constant	−0.0494***	−0.0580***	−0.0204***
	(−9.26)	(−9.51)	(−3.51)
ΔASYER		0.0255**	0.0581***
		(2.25)	(3.99)
lagΔdASYER		0.0217**	0.0165
		(2.15)	(1.61)
COVID	0.1859***	0.1756***	
	(19.21)	(16.39)	
ΔASYERXCOVID		0.1617***	
		(4.48)	
SOE	0.0222***	0.0234***	0.0252***
	(3.65)	(3.46)	(3.73)
ΔASYERXSOE			−0.0248
			(−1.20)
ΔSIZE	0.2258***	0.2313***	0.2367***
	(9.04)	(8.09)	(8.21)
ΔLEV	0.1525**	0.1997***	0.1961***
	(2.17)	(2.63)	(2.59)
ΔTANGIBILITY	0.3423***	0.3491**	0.3154**
	(3.31)	(2.54)	(2.32)
ΔTURNOVER	−0.0003	−0.0004***	−0.0005***
	(−1.57)	(−2.59)	(−2.72)
ΔMTB	−0.0133***	−0.0143***	−0.0180***
	(−3.55)	(−3.12)	(−3.60)
ΔCF	0.2363***	0.2990***	0.2586***
	(4.39)	(5.00)	(4.26)
ΔNWC	0.8486***	0.7758***	0.7820***
	(13.38)	(10.69)	(10.85)
ΔCAPX	−0.3114***	−0.2884***	−0.2911***
	(−4.20)	(−3.36)	(−3.39)
Industry_FE	Yes	Yes	Yes
Year_FE	NO	NO	Yes
N	8,020	5,596	5,596
adj. R2	0.199	0.215	0.210

This table presents the results of robustness checks of three models using changes in the variables. Model 1 and 2 control for industry fixed effect and Model 3 controls for industry×year fixed effect. The values in parentheses represent t-statistics. ***, **, and * denote statistical significance at the 1, 5, and 10% levels, respectively. Variables are defined in Table 1.

We further employ different measures of dependent variables to test the robustness of this study’s main findings. Pursuant to Gao et al. (2013), we use industry-adjusted measures of cash holdings (industry_adjusted_cash) to mitigate industry-specific characteristics. This variable is equal to the difference between the corporate and median industry cash holdings. Moreover, we use cash to net assets, which is widely used in prior research (Opler et al., 1999; Bates et al., 2009; Lei et al., 2018). The estimation results are presented in Tables 9 and 10. We find that the coefficients of ΔASYERXCOVID and COVID are all positive and statistically significant. The efficiency of ΔASYERXSOE remains insignificant. However, it still yielded the expected sign. Finally, we employ a two-step dynamic system generalized method of moments (GMM) to address the endogeneity issue. The estimates reported in Table 10 provide consistent evidence with the previous regressions.

**Table 9:** Robustness checks with alternative measures of dependent variables.

	indus_adjusted_cash	Ln (cash/net asset)
	MODEL 1	MODEL 2	MODEL 3	MODEL 1	MODEL 2	MODEL 3
Constant	−0.0972	−0.2361***	−0.2642***	−2.813^***^	−2.8326***	−2.9851***
	(−1.23)	(−2.96)	(−3.29)	(−20.64)	(−20.58)	(−21.98)
ASYER		0.0949***	0.1203***		0.0135	0.0100
		(9.39)	(8.99)		(0.77)	(0.48)
ASYERXCOVID		0.1120***			0.0035	
		(3.03)			(0.07)	
COVID	0.0242^**^	−0.0244		0.128^***^	0.1263***	
	(2.24)	(−1.50)		(8.04)	(5.24)	
SOE	0.0219^***^	0.0210***	0.0304***	0.0997^***^	0.0995***	0.0833***
	(2.97)	(2.86)	(3.35)	(7.77)	(7.75)	(5.14)
ASYERXSOE			−0.0365*			0.0292
			(−1.92)			(0.88)
SIZE	0.00865^***^	0.0135***	0.0148***	−0.00956^*^	−0.0089	−0.0013
	(2.62)	(4.06)	(4.42)	(−1.67)	(−1.55)	(−0.23)
LEV	−0.245^***^	−0.2685***	−0.2746***	0.282^***^	0.2780***	0.2430***
	(−6.96)	(−7.66)	(−7.83)	(4.97)	(4.90)	(4.24)
TANGIBILITY	0.0432	0.0452	0.0491	0.775^***^	0.7753***	0.8074***
	(1.04)	(1.09)	(1.18)	(11.99)	(11.99)	(12.47)
TURNOVER	0.0000268	0.0000	0.0000	0.000437^***^	0.0004***	0.0004***
	(0.37)	(0.23)	(0.34)	(4.75)	(4.74)	(4.75)
MTB	0.00558^*^	0.0053*	0.00312	0.00672	0.0066	0.0101**
	(1.77)	(1.70)	(0.96)	(1.47)	(1.45)	(2.21)
*CF*	−0.224^***^	−0.1033*	−0.0826	2.453^***^	2.4680***	2.3965***
	(−4.20)	(−1.95)	(−1.53)	(26.74)	(26.41)	(25.59)
NWC	0.454^***^	0.4750***	0.4723***	1.362^***^	1.3641***	1.3391***
	(13.42)	(14.09)	(14.04)	(25.60)	(25.63)	(24.95)
CAPX	−0.182^***^	−0.1946***	−0.2040***	−0.909^***^	−0.9104***	−0.9558***
	(−5.61)	(−6.05)	(−6.31)	(−16.27)	(−16.28)	(−17.03)
Industry_FE	Yes	Yes	YES	YES	YES	Yes
Year_FE	NO	NO	YES	NO	NO	Yes
N	11,415	11,415	11,415	11,411	11,411	11,411
adj. R2	0.156	0.167	0.167	0.375	0.375	0.371

This table presents the results of robustness checks of three models using alternative measures of dependent variables. Column (1)–(3) uses industry-adjusted measures of cash holdings (industry_adjusted_cash) to mitigate industry-specific characteristics, which is equal to the difference between the corporate and median industry cash holdings. Column (4)–(6) uses cash to net assets as the alternative measures of dependent variables. Model 1 and 2 control for industry fixed effect and Model 3 controls for industry×year fixed effect. The values in parentheses represent t-statistics. ***, **, and * denote statistical significance at the 1, 5, and 10% levels, respectively. Variables are defined in Table 1.

**Table 10:** Robustness checks using two-step GMM.

	Two-step GMM
	MODEL 1	MODEL 2	MODEL 3
Constant	−1.017	0.179	−0.632***
	(−0.66)	(0.22)	(−2.02)
L.CASH	0.0671	0.0245	−0.279***
	(0.23)	(0.40)	(−7.26)
ASYER		0.0386***	0.311***
		(2.93)	(4.41)
ASYERXCOVID		0.248***	
		(4.52)	
COVID	0.138***	0.0498*	
	(4.57)	(1.90)	
SOE	0.393	0.125***	0.283***
	(1.24)	(2.63)	(4.64)
ASYERXSOE			−0.643***
			(−3.72)
SIZE	−0.0229	0.0508	0.0410***
	(−0.39)	(1.49)	(3.51)
LEV	−2.007***	−1.103**	−0.262
	(−2.69)	(−2.50)	(−1.52)
TANGIBILITY	3.438*	−0.830*	0.0551
	(1.74)	(−1.90)	(0.32)
TURNOVER	−0.00235	0.00116	0.000182
	(−0.97)	(0.89)	(1.12)
MTB	−0.00544	0.0289*	0.0645**
	(−0.30)	(1.91)	(2.12)
*CF*	0.0762	0.0193	−0.315***
	(0.54)	(0.21)	(−3.62)
NWC	−1.282	0.326	0.310*
	(−1.38)	(1.30)	(1.93)
CAPX	−0.801	0.162	−0.408***
	(−0.99)	(0.49)	(−2.81)
AR (2)	0.875	0.268	0.238
Hansen J test	0.673	0.111	0.326

This table presents the results of robustness checks of three models using two-step GMM to solve the endogeneity issues. The values in parentheses represent t-statistics. ***, **, and * denote statistical significance at the 1, 5, and 10% levels, respectively. Variables are defined in Table 1.

Collectively, the robustness checks carried out in the subsection yield similar results as before. However, while not all regressions are statistically significant, they had their expected signs. First, COVID-19 is positively related to firms’ cash holdings. Furthermore, information asymmetry has a stronger impact on corporate cash holdings during the COVID-19 pandemic. Finally, the information asymmetry’s effect on corporate cash holdings is weaker for SOEs.

## Conclusion

Despite the existing theoretical connection concerning the relationship between information asymmetry and cash holdings, scant research has been conducted on how other factors affect their relationship. Accordingly, we extended the analysis by introducing state-ownership heterogeneity and the impact of an exogenous shock (i.e., the COVID-19 pandemic) into the picture. The present study theoretically and empirically investigated how state ownership and the COVID-19 pandemic affected the relationship between information asymmetry and corporate cash holdings. To do this, we used samples of Chinese listed firms from 2014 to 2020. Through this study, we provided evidence showing that COVID-19 is positively related to Chinese listed firms’ cash holdings. Additionally, we showed that information asymmetry exerts a stronger effect on corporate cash holdings during the COVID-19 pandemic. We further illustrated that information asymmetry has a weaker effect on corporate cash holdings for SOEs, even during the COVID-19 pandemic. In other words, state ownership moderates information asymmetry’s impact on cash holdings and softens firms’ precautionary motives for cash holdings under the COVID-19 pandemic. Finally, we employed yearly changes in dependent variables and various measures of dependent variables to conduct additional analyses. This helped in alleviating endogeneity issues and corroborating the results. Overall, our analysis suggests that information asymmetry exerts a stronger effect on cash holdings under the impact of COVID-19. However, such an effect is weaker for SOEs than for non-SOEs.

The cash held by firms in various countries has been rising in the decades before the COVID-19 pandemic. Moreover, firms do not appear to have been taking the suggestions to use cash for repurchasing or increasing investments. As we speak, it remains difficult to conclude that the virus is already in control. In the long run, firms’ saving behaviors may be strengthened because of the influence of the COVID-19 shock. This may be brought about by the reinforcement of their precautionary motives. Our results from the Chinese sample suggest that firms use cash to repay their debt, increase dividends, and invest in economic recovery. It is somehow dependent on what firms do with their cash balances. This determines the picture of the economy’s future. Furthermore, this study implies that Chinese policy makers should have more efficient and productive support to channel funds to non-SOEs, especially when the economy is involved in exogenous shocks such as the COVID-19 pandemic. State ownership tends to support corporate stability even though it is also inclined to soften firms’ precautionary motive for cash holdings. This is true particularly in cases such as the COVID-19 pandemic. On this basis, the policy should ensure that the Chinese government is serving to improve information transparency instead of exacerbating information asymmetry to take advantage of their privileged access to information at other investors’ expense. Moreover, our study calls for more information transparency-related policies in Chinese firm disclosure. This will improve the information environment not only to benefit firms’ cash policies but also to contribute to the capital market’s functioning.

The economic behavior of a company in a developing country with weaker governance regimes may be influenced by a variety of factors (Latif et al. 2022). For SOEs in China, corporate governance is complicated due to agency problems among the state, listed firms, and SOE managers. As a result, the corporate governance of firms is a hybrid of Western and Chinese characteristics (Beck and Brodsgaard, 2022). Effective corporate governance mechanisms must achieve a balance between different parties in order to improve the efficiency and performance of the state sector’s management. While it is true that supporting SOEs to ensure that basic public services are provided is crucial in an emergency, the Chinese government must consider the implications of such measures on the market’s dynamics. In times of credit scarcity, channeling state resources to SOEs can exacerbate the risk of a potential financial crisis and delay the recovery process. Therefore, the Chinese government should provide financial support to firms which are competitive and in urgent demand, instead of simply prioritizing them on the basis of whether they are SOEs or non-SOEs.

Finally, future research could extend the analysis to other markets, regions, types of ownership, such as foreign ownership. Additionally, institutional environment and legal systems could be considered for further information asymmetry-cash holding nexus study.

## Data availability statement

The original contributions presented in the study are included in the article/supplementary material, further inquiries can be directed to the corresponding author.

## Author contributions

DY: conceptualization, validation, investigation, writing-original draft, and visualization. WS: writing review and editing, conceptualization, and validation. BN and MHY: analysis, corrections, and revisions. BL: revisions, editing, and proofreading. All authors contributed to the article and approved the submitted version.

## Funding

This study was funded by a grant from Universiti Putra Malaysia, No. 9696800.

## Conflict of interest

The authors declare that the research was conducted in the absence of any commercial or financial relationships that could be construed as a potential conflict of interest.

## Publisher’s note

All claims expressed in this article are solely those of the authors and do not necessarily represent those of their affiliated organizations, or those of the publisher, the editors and the reviewers. Any product that may be evaluated in this article, or claim that may be made by its manufacturer, is not guaranteed or endorsed by the publisher.

## Footnotes

^1^Source: WIND database.

^2^All unreported regression results are available on request from the author.

^3^The result is calculated as coefficient of COVID dummy ÷ mean value of cash ratio (CASH).

^4^The marginal effect is calculated as coefficient of information asymmetry (ASYER) + coefficient of interaction term (ASYERXCOVID). If COVID = 0, then the result equal to the coefficient of information asymmetry (ASYER).

^5^The result is calculated as coefficient × standard deviation of information asymmetry (ASYER), deflated by the mean value of the cash ratio (CASH).
